# Matrin 3 Binds and Stabilizes mRNA

**DOI:** 10.1371/journal.pone.0023882

**Published:** 2011-08-17

**Authors:** Maayan Salton, Ran Elkon, Tatiana Borodina, Aleksey Davydov, Marie-Laure Yaspo, Eran Halperin, Yosef Shiloh

**Affiliations:** 1 The David and Inez Myers Laboratory for Cancer Research, Department of Human Molecular Genetics and Biochemistry, Sackler School of Medicine, Tel Aviv University, Tel Aviv, Israel; 2 Division of Gene Regulation, The Netherlands Cancer Institute, Amsterdam, The Netherlands; 3 Max-Planck-Institute for Molecular Genetics, Berlin-Dahlem, Germany; 4 Department of Molecular Microbiology and Biotechnology, George S. Wise Faculty of Life Sciences, Tel Aviv University, Tel Aviv, Israel; The Rockefeller University, United States of America

## Abstract

Matrin 3 (MATR3) is a highly conserved, inner nuclear matrix protein with two zinc finger domains and two RNA recognition motifs (RRM), whose function is largely unknown. Recently we found MATR3 to be phosphorylated by the protein kinase ATM, which activates the cellular response to double strand breaks in the DNA. Here, we show that MATR3 interacts in an RNA-dependent manner with several proteins with established roles in RNA processing, and maintains its interaction with RNA via its RRM2 domain. Deep sequencing of the bound RNA (RIP-seq) identified several small noncoding RNA species. Using microarray analysis to explore MATR3′s role in transcription, we identified 77 transcripts whose amounts depended on the presence of MATR3. We validated this finding with nine transcripts which were also bound to the MATR3 complex. Finally, we demonstrated the importance of MATR3 for maintaining the stability of several of these mRNA species and conclude that it has a role in mRNA stabilization. The data suggest that the cellular level of MATR3, known to be highly regulated, modulates the stability of a group of gene transcripts.

## Introduction

Matrin 3 (MATR3) is a highly conserved, inner nuclear matrix protein of 125 kDa [1]. Nuclear matrix proteins bound to the inner nuclear membrane form a skeletal nuclear framework with roles in chromatin organization, DNA replication, transcription, repair, and RNA processing and transport [2]. MATR3 contains a bipartite nuclear localization signal (NLS) [3], two zinc finger domains predicted to bind DNA, and two RNA recognition motifs (RRM). Rat MATR3 was shown to bind DNA [4], [5], but an RNA binding activity was never attributed to MATR3. A missense mutation in a domain-less area on MATR3 was recently found to cause adult-onset autosomal dominant vocal cord and pharyngeal weakness with distal myopathy (VCPDM) [6]. Together with the proteins SFPQ (PSF) and NONO (p54nrb), MATR3 has been implicated in the nuclear retention of hyper-edited mRNA, which prevented its translation [7]. Recently we found MATR3 to be phosphorylated in response to the induction of double strand breaks in the DNA. This phosphorylation depended the nuclear protein kinase ATM. Notably, SFPQ and NONO were also implicated in the DNA damage response in that study [8].

MATR3 was reported to be phosphorylated by the protein kinase PKA following activation of the NMDA receptors, which led to its degradation [9]. A proteomic screen revealed that MATR3 binds to calmodulin and it was suggested that it is cleaved by both caspase-3 and caspase-8 [10]. MATR3 levels decrease after treatment with soy extract of homocysteine-stressed endothelial cells [11], and are reduced in the brain of Down syndrome fetuses [12]. It has recently been suggested that the microRNA miR-200b, which may be involved in massive macronodular adrenocortical disease (MMAD), modulates MATR3 cellular amounts [13]. However, taken together these studies do not point at a specific role for MATR3 in cellular metabolism.

Following our earlier observation of MATR3′s involvement in the DNA damage response [8], we set out to further explore its cellular functions. Proteomic analysis revealed a protein-RNA complex containing MATR3 together with other RNA metabolizing enzymes, whose integrity was RNA-dependent. Identification of several RNA species in this complex points to its involvement in RNA processing. Our data further suggest that MATR3 contributes to stabilizing certain mRNA species.

## Results

### MATR3 maintains an RNA-dependent association with proteins involved in RNA processing

**Figure 1:**
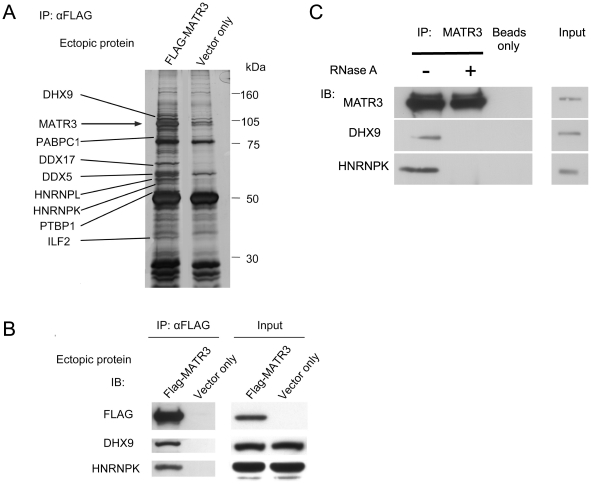
Identification of novel MATR3 interactors. (**A**) FLAG-MATR3 was expressed in HEK293T cells and immunoprecipitated using anti-FLAG conjugated beads. Immune complexes were separated by SDS-PAGE and visualized with silver staining. Cells with empty vector allowed discrimination between specific and non-specific immunoprecipitation. Bands that appeared specific were identified using mass spectrometry. (**B**) FLAG-MATR3 was expressed in HEK293T cells and immunoprecipitated using FLAG-conjugated beads. The immune complexes were blotted with the indicated antibodies. (**C**) Endogenous MATR3 was immunoprecipitated from HEK293T cells, and the immune complexes were treated with RNase A and blotted with antibodies against the indicated proteins.

In order to obtain clues to MATR3′s function we set to identify novel MATR3-interacting proteins. FLAG-tagged MATR3 was expressed in HEK293T cells and immunoprecipitated using anti-FLAG antibody, with an empty vector serving as control. The immune complexes were separated using SDS-PAGE, and the gels underwent silver staining (Fig. 1A). Bands that came down with MATR3 were identified using mass spectrometry.

Individual interactions with MATR3 were validated by co-immunoprecipitation. The proteins DHX9 and HNRNPK co-immunoprecipitated with ectopic and endogenous MATR3 (Fig. 1B and C). In view of the RRM domains in MATR3 we suspected that some of these interactions might require RNA molecules. RNase treatment indeed abolished MATR3′s interactions with DHX9 and HNRNPK (Fig. 1C), suggesting that RNA was necessary for maintaining these interactions.

### MATR3 binds RNA via its RRM2 domain

**Figure 2:**
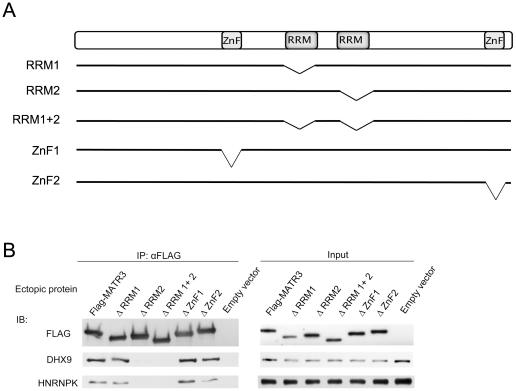
MATR3 binds RNA via its RRM2 domain. (**A**) Schematic presentation of MATR3 domains and deletion of specific domains in different constructs used in this study. (**B**) HEK293T cells were transfected with the indicated MATR3 expression constructs. FLAG-conjugated beads were used for immunoprecipitation and the immune complexes were blotted with the indicated antibodies.

MATR3 has two zinc finger domains that can potentially interact with DNA, and two RRMs that are known to interact with RNA (Fig. 2A). These domains are common to nuclear matrix proteins, underscoring their involvement in transcription and RNA processing. Indeed, MATR3′s interactions with DHX9 and HNRNPK were RNA-dependent (Fig. 1C). We examined the contribution of the RRM domains to MATR3′s ability to bind RNA and its interactors by preparing constructs expressing MATR3 with deletions of one or both of its RRMs, or one of the zinc finger domains (Fig. 2A). Notably, MATR3′s ability to bind the proteins DHX9 and HNRNPK depended on the presence of RRM2. Deletion of the RRM1 and ZnFn2 domains had a moderate effect on these interactions (Fig. 2B).

### Analysis of RNA species associated with the MATR3 complex

Co-immunoprecipitation of MATR3 with its interactors was RNA- and RRM2-domain-dependent, suggesting that MATR3 binds RNA molecules via its RRM2 domain, and this RNA is important for MATR3 interactions. We undertook to identify these RNA species. FLAG-MATR3 was immunoprecipitated from HEK293T cells, and RNA was extracted from the immune complexes and underwent RNA-seq using the Illumina/Solexa technology. We used total cellular RNA as background control. While an ideal control would have been RNA obtained from immune complexes of RRM2-deleted MATR3, the amount of RNA found in such immune complexes was minute and did not allow sequencing. We thus identified 4 RNA species in MATR3 immune complexes that were significantly over-represented in these complexes compared to their occurrence in total cellular RNA. All of these RNAs turned out to be small noncoding RNAs (Table 1).

**Table 1:** Small noncoding RNAs identified in MATR3 immunoprecipitates.

Designation	Accession no.	Documented function
U4	NR_003925	mRNA splicing [36].
SNORA73A	NR_002907	Possible role in rRNA proccesing [37], [38].
7SK	NR_001445	Inhibition of transcription elongation [25].
RMRP	NR_003051	rRNA processing and endonuclease activity [39].

**Figure 3:**
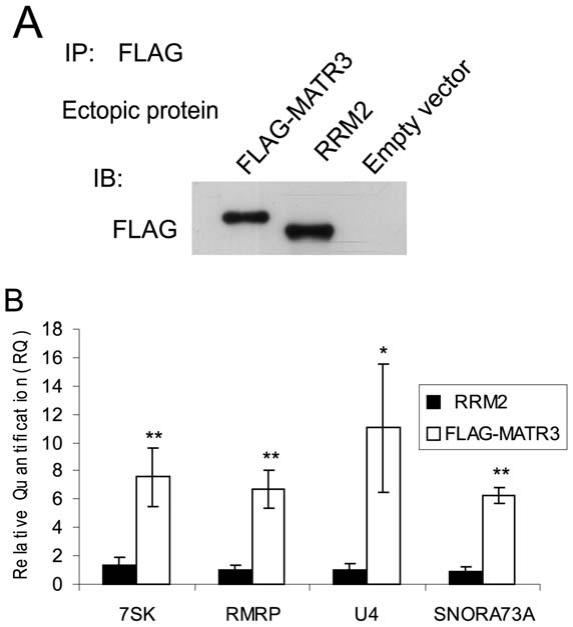
Validation of RIP-seq results. (**A**) HEK293T cells were transfected with the indicated MATR3 constructs and the ectopic MATR3 proteins were immunoprecipitated using FLAG-conjugated beads and blotted with the indicated antibodies. (**B**) RNA was extracted from the immune complexes, yeast Phe-tRNA was added, qPCR was carried out on reverse transcription products and the Relative Quantification (RQ) is shown as fold-change of signal compared to ΔRRM2 minus the background of the empty vector. The plot represents the mean of three independent experiments and error bars represent SD (*P≤0.05, **P≤0.01, t test).

The deep sequencing results were validated using qPCR. FLAG-MATR3, FLAG-MATR3 ΔRRM2 and the empty vector were expressed in HEK293T cells, were immunoprecipitated using FLAG-conjugated beads, and RNA was extracted from the immune complexes. Phe-tRNA from *S. Cerevisiae* was added to this RNA as an exogenic control. The results confirmed that all 4 small noncoding RNAs co-immunoprecipitated with wild type MATR3 (Figs. 3A and B).

### Effect of MATR3 loss on the cellular transcriptome

**Figure 4:**
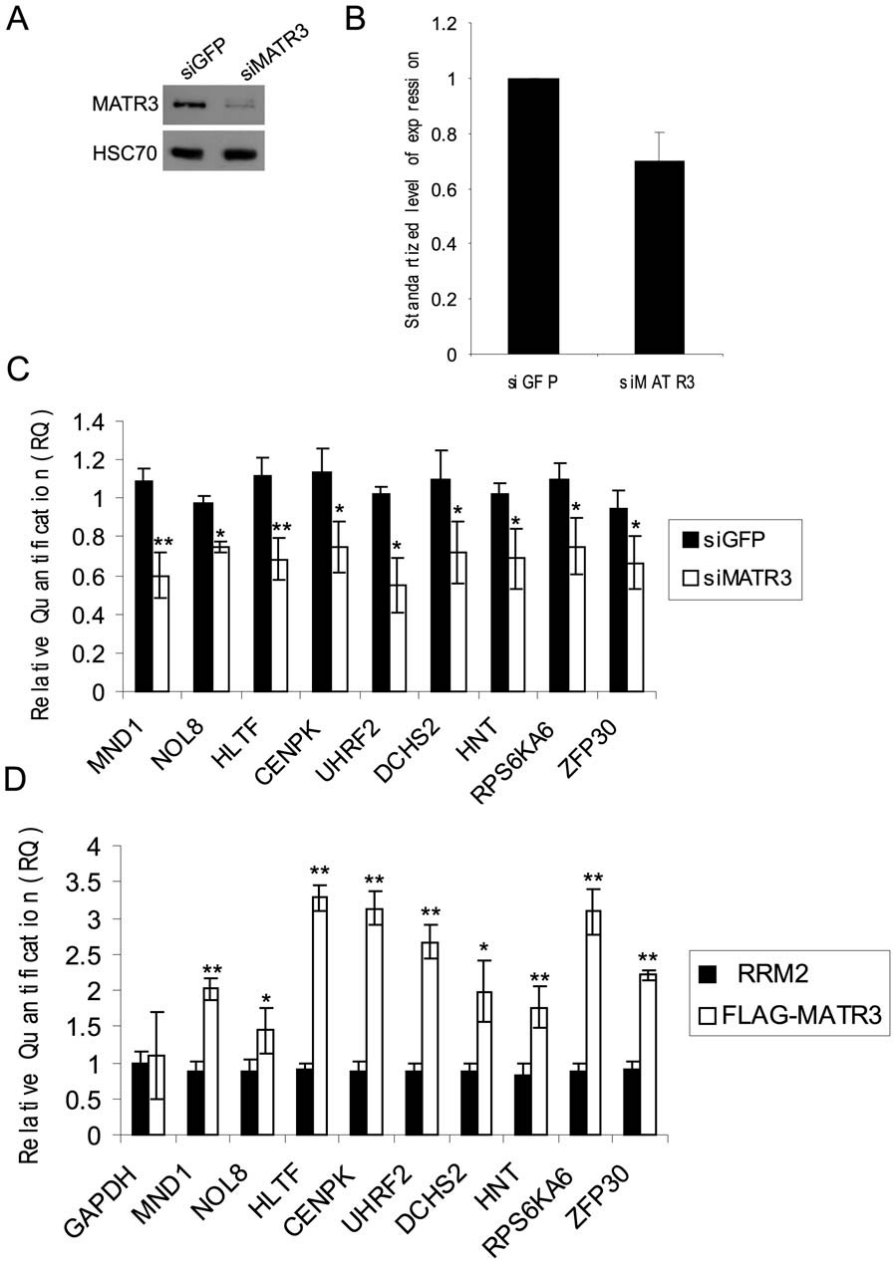
MATR3 affects the transcript level of mRNAs that bind to it. (**A**) Knockdown of MATR3 in U2OS cells: western blotting analysis of total cellular extracts 96 hr after transfection with siGFP or siMATR3. (**B**) Effect of MATR3 depletion on the expression of 77 human genes. Expression profiles were recorded in U2OS cells knocked-down for MATR3 and in control cells transfected with siGFP, using Affymetrix Human Gene 1.0 ST arrays. Responding genes in the datasets (defined as showing at least 1.7 fold-change in expression level) underwent clustering analysis using the CLICK algorithm [35]. A cluster containing 77 genes was obtained containing genes whose expression level was reduced in MATR3 knocked-down cells (**Table S1**). The plot represents average expression levels of these genes normalized against their average level of expression in the siGFP samples. (**C**) Validation of results obtained using microarray analysis. RNA was extracted from U2OS cells knocked-down for MATR3 and from control cells transfected with siGFP was reverse transcribed, and qPCR was carried out. Shown is the relative quantification (RQ) as fold-change compared to the siGFP cells. Two endogenous control transcripts were used: GAPDH and hTBP. The plot represents the mean of three independent experiments and error bars represent SD (*P≤0.05, **P≤0.01, t test). (**D**) Binding of mRNAs to MATR3. The extent of binding of mRNAs to wild type vs. ΔRRM2 served as a measure of mRNA binding to the protein. RNA was extracted from the MATR3 immune complexes shown in Figs. 3A and Phe-tRNA was added. qPCR was carried out on reverse transcription products and the Relative Quantification (RQ) is shown as fold-change of signal compared to ΔRRM2 minus the background of the empty vector. The plot represents the mean of three independent experiments and error bars represent SD (*P≤0.05, **P≤0.01, t test).

Since the small noncoding RNA 7SK and the protein DHX9 are involved in transcription regulation [14], [15], [16], [17], [18] and HNRNPK is a co-activator of p53 [19], we asked whether MATR3 depletion would affect the cellular transcriptome. We depleted U2OS cells of MATR3 using RNAi (Fig. 4A) and examined the effect on gene expression patterns using microarray analysis. While MATR3 was not required for transcription of p53 target genes (Fig. S1), we identified a cluster of 77 genes whose expression levels were reduced in MATR3-depleted cells (Fig. 4B and Table S1). These results were validated for 9 transcripts using qPCR . Indeed, the levels of all 9 transcripts were reduced following MATR3 depletion to 55–75% of their levels in control cells (Fig. 4C).

### MATR3 binds mRNAs

We asked whether MATR3 maintained physical interaction with mRNAs whose levels were reduced following its depletion. Such mRNAs may not have been over-represented initially in MATR3 immunoprecipitates due to their low abundance. Using qPCR we examined specifically the presence of 9 mRNAs of this group in MATR3 immunoprecipitates, with GAPDH mRNA serving as control. Importantly, all mRNAs were found to bind wild type but not ΔRRM2-MATR3 (Fig 4D). These results suggest that MATR3 interacts physically with specific transcripts whose levels are affected by its presence.

### MATR3 affects mRNA stability

**Figure 5:**
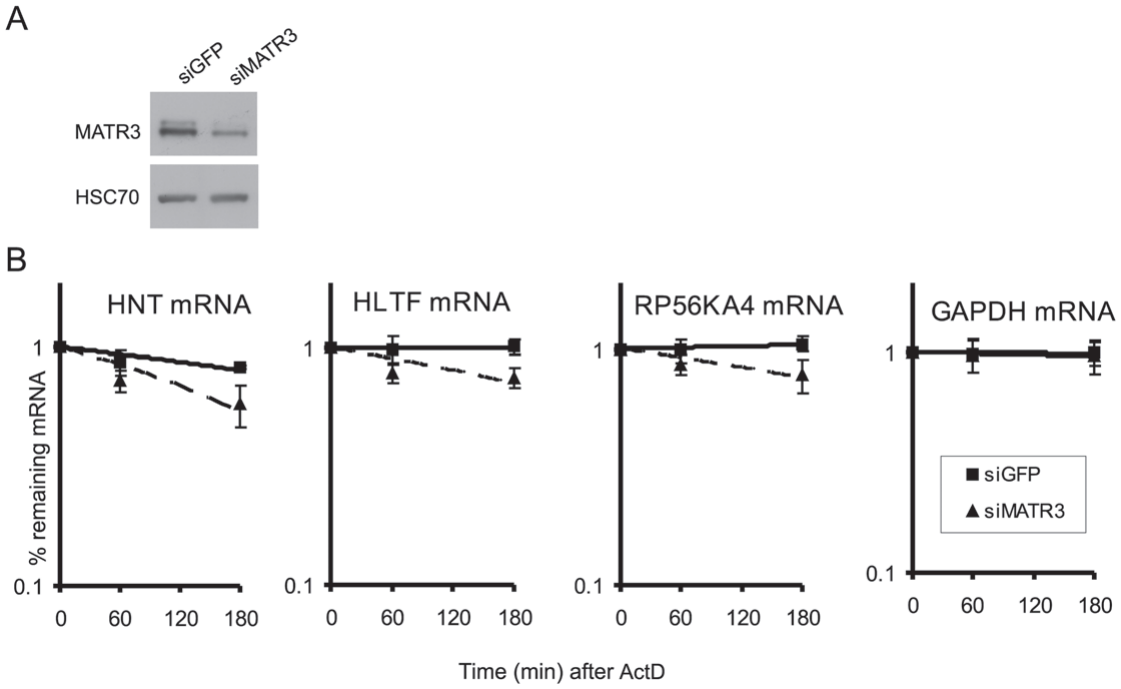
MATR3 stabilizes mRNAs. (**A**) Western blotting of total cellular extracts showing the extent of MATR3 knockdown in U2OS cells 96 hr after transfection with siGFP or siMATR3. (**B**) mRNA half-life was estimated by treating siGFP cells and siMATR3 cells with actinomycin D (2 µg/ml) for the indicated time periods. HLTF, RP56KA4, HNT and GAPDH mRNA levels were measured using qPCR, normalized against 18S rRNA levels and plotted on a scale. The plot represents the mean of three independent experiments and error bars represent SD.

In view of the interaction between MATR3 and mRNAs whose amounts were affected by its depletion, we asked whether MATR3 is involved in maintaining the stability of these mRNAs. We measured the half-life of 3 mRNAs in this group (HLTF, RP56KA4, HNT) in cells proficient or deficient of MATR3, by monitoring the decay of these mRNAs after inhibition of de novo transcription using actinomycin D. Indeed, MATR3 depletion reduced the stability of the 3 mRNAs compared to GAPDH mRNA (Fig. 5). This reduction in stability could account for the decrease in the amounts of these mRNAs following MATR3 depletion.

## Discussion

MATR3′s activity and mode of action are unclear, but its domains predict a role in RNA metabolism. We identified DHX9 and HNRNPK as new interactors of MATR3. DHX9 is a DNA and RNA helicase with diverse physiological functions in transcription, RNA processing, transport [20] and translation [21]. HNRNPK, a component of the heterogeneous nuclear ribonucleoprotein complex, is involved in chromatin remodeling and mRNA transcription, splicing and translation [22].

In view of the involvement of MATR3′s RRM domains and its new interactors in RNA metabolism, we asked whether RNA is involved in these interactions and found them to be dependent on both RNA and the RRM2 domain of MATR3. Furthermore, we obtained a first demonstration that MATR3 is an RNA-binding protein. Deep sequencing of bound RNA identified several small noncoding RNAs, which were over-represented in MATR3 immunoprecipitates and whose binding depended on MATR3′s RRM2 domain. Interestingly, one of these RNAs was 7SK, which is known to bind HNRNPK [14], [23], [24], [25], [26], [27] and DHX9 proteins [23]. 7SK is a regulator of the P-TEFb kinase, which phosphorylates RNA polymerase II to promote transcription elongation [28], [29]. Thus, our data point to a protein complex containing MATR3, DHX9 and HNRNPK and the 7SK RNA. It is known that depletion of HNRNPK changes 7SK's interaction with its surrounding proteomic environment [24]. In our experiments, depletion of MATR3 did not exert a similar effect (data not shown).

While HNRNPK was shown to co-activate p53, our microarray analysis indicated that MATR3 is not involved in p53 activation (Fig. S1). However, MATR3 depletion led to decreased amounts of 77 mRNAs (Fig. 4A). MATR3 binding of 9 transcripts of this group (Fig. 4C) was further validated (Fig. 4D), and further experiments suggested that MATR3 is involved in controlling the levels of these transcripts by affecting their stability (Fig. 5).

The half-life of mRNA molecules is affected by specific sequences usually located in the 3′ UTR, and is regulated by RNA-binding proteins that bind to these sequences (48,49). An extensively documented example is the ARE sequence, a short sequence (AUUUA) found in the 3′ UTR of many mRNAs (50,51). RNA-binding proteins such as the Hu proteins bind to this sequence and enhance the stability of the corresponding mRNAs (52,53). The steady-state levels of the mRNAs thus depend on the balance between their own production and the levels and activity of proteins that stabilize them or enhance their degradation.

The levels of cellular MATR3 are highly regulated [9], [10], [11], [12], [13]. We suggest that this tightly regulated protein stabilizes a number of transcripts, probably via direct interaction with these RNA species. We also propose that MATR3 is part of a protein complex containing, among others, the DHX9 and HNRNPK proteins as well as small noncoding RNAs such as 7SK. DHX9 and HNRNPK as well as the 7SK RNA were previously found to be involved in transcription and several RNA processes such as splicing [22], [30], [31]. Identification of MATR3 as another player in this complex might shed light on new roles of MATR3 in RNA metabolism.

## Materials and Methods

### Cell lines

HEK293T (ATCC Number: CRL-11268) and U2OS (ATCC Number: HTB-96) cells were grown in DMEM supplemented with 10% fetal bovine serum, at 37°C and 5% CO_2_ atmosphere.

### Antibodies and other reagents

Polyclonal antibodies against MATR3, DHX9, and HNRNPK were obtained from Bethyl Laboratories (Montgomery, TX). FLAG-conjugated beads were purchased from Sigma-Aldrich, RNase A from RBC (Taipei, Taiwan), neocarzinostatin from KayaKU Chemicals (Tokyo, Japan) and actinomycin D from Sigma-Aldrich.

### Expression constructs

A full-length cDNA clone of MATR3, KIAA0723, was obtained from the Kazusa DNA Research Institute (Kisarazu, Japan) and cloned into pCMV:FLAG2B vector. Deletions in the cDNA were generated by Pfu polymerase amplification using the MATR3 construct as template and primers flanking the domain to be deleted.

### Immunoblotting and immunoprecipitation

Immunoblotting and immunoprecipitation were carried out according to standard techniques. Briefly, cells were harvested and lysed in RIPA lysis buffer, and the lysates were run on 8% SDS PAGE and transferred onto a nitrocellulose membrane. For immunoprecipitation, cells were washed twice with ice-cold PBS, harvested, and lysed for 30 min on ice in 0.5% NP40, 150 Mm NaCl, 50 Mm Tris pH7.5, and 1 mM EDTA supplemented with a mixture of protease and phosphatase inhibitors. Supernatants were collected and the primary antibody was added for 2 hr at 4°C. Protein A and G sepharose beads were added for an additional 1 hr, after which the beads were washed 4 times. Beads were boiled in sample buffer and loaded onto the gel. In the RNA-IP experiment the RNA was extracted from the immune complexes after the above IP procedure. Mass spectrometric analysis was carried out as previously described [32].

### RNAi

RNA duplexes of 19 nucleotides (AGACTTCCATGGACTCTTA) targeting human MATR3 mRNA were designed, and subsequently synthesized by Dharmacon (Lafayette, CO) with the OnTarget Plus modifications. The above oligonucleotide was used for the microarray experiment and subsequent experiments were carried out using OnTarget Plus SMARTpool against MATR3, which was obtained from Dharmacon (Lafayette, CO). U2OS cells were grown to 20%–50% confluency and transfected with siRNA using the DharmaFECT 1 reagent (Lafayette, CO).

### RNA purification

RNA was isolated from cells or immune complexes using the RNeasy plus mini kit (QIAGEN).

### RNase treatment

Following protein immunoprecipitation, immune complexes bound to beads were washed twice with lysis buffer containing 0.5% NP-40 and suspended in the same buffer containing 0.1 mg/ml of RNase A for 15 min at room temperature.

### Preparation of libraries for Illumina sequencing platform

Libraries were prepared as described in Sultan et al. 2008 [33] with the following modifications: just before library amplification, uridine digestion was performed at 37°C for 15 min in 5 µl of 1xTE buffer, pH 7.5, with 1 U of UNG (Applied Biosystems, Foster City, CA); different ligation adapters and PCR primers were used (for paired-end sequencing, Illumina kit #PE-102-1002).

### Solexa sequencing

The SOAP program [34] was used to align the sequence reads to genomic sequences. Reads containing mismatches to genomic sequences aligning to multiple genomic positions were disregarded. For the remaining reads, we searched for genomic positions aligning to at least 5 reads (p value 10̂-15 under a Poisson distribution). For each gene that contained one of these positions we counted the overall number of distinct positions with at least one aligned read in the gene. To avoid sequencing artifacts we removed genes that had 10 different aligned reads at most. This process resulted in a list of 60 genes that were manually inspected.

### Quantitative real-time RT–PCR

cDNA synthesis was carried out with the High Capacity cDNA Reverse Transcription Kit (Applied Biosystems). qPCR was performed with the Power SYBR Green RT-PCR Master Mix (Applied Biosystems) and the ABI PRISM 7900HT sequence detection system (Applied Biosystems). The comparative Ct method was employed to quantify transcripts, and delta Ct was measured in triplicate.

RIP-Sequencing results were normalized against *S. Cerevisiae* Phe-tRNA (Sigma-Aldrich), which was added to the samples after RNA extraction of the immune complexes. Primers used in the RT–PCR assays are provided in Table S2.

### mRNA half-life

U2OS cells were transfected with siGFP (irrelevant siRNA) or siRNA against MATR3, and 96 hr later the cells were treated with actinomycin D (2 µg/ml) for different time points, and harvested in Trizol reagent (Sigma-Aldrich). Total RNA was used for qPCR. For each time point, amounts of mRNAs were normalized against 18S rRNA and half-lives were calculated relative to untreated sample.

## Supporting Information

Figure S1 **Expression profiles were recorded in U2OS cells knocked-down for MATR3 and in control cells transfected with siGFP, using the Affymetrix Human Gene 1.0 ST arrays.** Profiles were measured at two time-points (3 and 6 hr) after treatment with the radiomimetic drug neocarzinostatin (NCS) and in time-matched untreated controls. Responding genes in the datasets (defined as those showing at least 1.7 fold-change in expression level following NCS treatment) were subjected to clustering analysis by the CLICK algorithm [35]. Cluster #1 contains the genes that were induced by NCS treatment. Known targets of p53 (e.g., p21, Mdm2, Fas, Gdf15, Apaf1) appear in this cluster.
(EPS)

Table S1 **77 genes whose expression levels were reduced in MATR3-depleted cells.**
(XLS)

Table S2 **Primers used for real-time PCR.**
(XLS)
